# Impacts of Embryonic Thermal Programming on the Expression of Genes Involved in *Foie gras* Production in Mule Ducks

**DOI:** 10.3389/fphys.2021.779689

**Published:** 2021-12-03

**Authors:** William Massimino, Charlotte Andrieux, Sandra Biasutti, Stéphane Davail, Marie-Dominique Bernadet, Tracy Pioche, Karine Ricaud, Karine Gontier, Mireille Morisson, Anne Collin, Stéphane Panserat, Marianne Houssier

**Affiliations:** ^1^Univ Pau & Pays Adour, INRAE, E2S UPPA, UMR 1419, Nutrition, Métabolisme, Aquaculture, Saint-Pée-sur-Nivelle, France; ^2^Univ Pau & Pays Adour, E2S UPPA, IUT Génie Biologique, Mont-de-Marsan, France; ^3^UEPFG INRAE Bordeaux-Aquitaine, Domaine d’Artiguères 1076, Benquet, France; ^4^GenPhySE, Université de Toulouse, INRAE, ENVT, Castanet-Tolosan, France; ^5^BOA, INRAE, Université de Tours, Nouzilly, France

**Keywords:** embryonic thermal programming, liver, metabolism, gene expression, duck

## Abstract

Embryonic thermal programming has been shown to improve *foie gras* production in overfed mule ducks. However, the mechanisms at the origin of this programming have not yet been characterized. In this study, we investigated the effect of embryonic thermal manipulation (+1°C, 16 h/24 h from embryonic (E) day 13 to E27) on the hepatic expression of genes involved in lipid and carbohydrate metabolisms, stress, cell proliferation and thyroid hormone pathways at the end of thermal manipulation and before and after overfeeding (OF) in mule ducks. Gene expression analyses were performed by classic or high throughput real-time qPCR. First, we confirmed well-known results with strong impact of OF on the expression of genes involved in lipid and carbohydrates metabolisms. Then we observed an impact of OF on the hepatic expression of genes involved in the thyroid pathway, stress and cell proliferation. Only a small number of genes showed modulation of expression related to thermal programming at the time of OF, and only one was also impacted at the end of the thermal manipulation. For the first time, we explored the molecular mechanisms of embryonic thermal programming from the end of heat treatment to the programmed adult phenotype with optimized liver metabolism.

## Introduction

The concept of early life programming appeared in humans less than 30 years ago with the observation of an association between the size of a child at birth and the risk of developing chronic pathologies in adulthood (Barker et al., 1993). More recently, programming has emerged in farm animals as an effective technique for improving their performances as adults. The principle consists in exposing the organism, during an early period of its life, characterized by a great plasticity, to an environmental stimulus which can thus be “recorded” and which can direct an adapted response to another stimulus, occurring later in its life (Lucas, 1998). In mammals, several types of primary stimulus have been associated to an alternative phenotype in adulthood such as developmental malnutrition, stress or hypoxia (Tarry-Adkins and Ozanne, 2011).

In birds, since embryogenesis occurs outside the mother’s body, many types of environmental factors can be easily applied to “program” the animals. Incubation temperature being one of the easiest parameters to control, embryonic thermal programming is therefore one of the most studied in poultry and has already demonstrated its efficiency in terms of improving adult thermoregulation in multiple species such as chickens (Piestun et al., 2008), ducks (DuRant et al., 2013) or quails (Vitorino Carvalho et al., 2020). Interestingly, changing the incubation temperature can also improve other performances, such as meat production in chicken (Piestun et al., 2013), or even *foie gras* production in mule duck (Massimino et al., 2019). In this last study, our team demonstrated that three different conditions of embryonic thermal programming led to an increase in lipid content in the liver of adult mule ducks after overfeeding (OF) (Massimino et al., 2019). A parallel study, focusing on the ontogeny of mule duck liver (Massimino et al., 2020), demonstrated that these thermal manipulations occurred during a period characterized by high expression of carbohydrate and lipid metabolism genes, suggesting a greater sensitivity of these metabolic pathways to thermal stress applied during this period. Nevertheless, despite the many encouraging results, the molecular mechanisms of programming process remain largely unknown. Modification of hormonal responses, gene expression regulations or epigenetic marks have been studied in the context of increased thermoregulation (Vaiserman et al., 2018; Madkour et al., 2021), but the field concerning hepatic metabolism has been scarcely explored (Loyau et al., 2014).

Overfeeding is a method used to stimulate *de novo* lipogenesis in migratory birds that store their energy in the liver (Leveille et al., 1975). For about 12 days, the ducks are fed corn (grain or meal) twice a day and the excess carbohydrates are converted into lipids by the hepatocytes, resulting in a huge increase in liver weight (Baeza et al., 2013). The metabolic pathways involved in this hepatic steatosis have been more precisely described in the last decades (Herault et al., 2010). First, glucose is taken up by the liver (Annabelle et al., 2017), before being degraded into pyruvate and then into acetyl CoA which is subsequently used for lipid synthesis (Annabelle et al., 2018). These lipids can be transported to peripheral tissues or recaptured by the liver for long-term storage (Tavernier et al., 2017).

The embryonic thermal programming applied to mule ducks that resulted in the increase in liver fattening (Massimino et al., 2019) may have affected each of these metabolic pathways, but also a variety of other pathways such as cell proliferation or thyroid hormone pathway strongly involved in liver metabolism (Sinha et al., 2014). In this study, we examined for the first time the short and long term molecular impacts of a 1°C increase in incubation temperature applied discontinuously (16 h/24 h) between E13 and E27 on mule duck eggs, resulting in the best optimized response to overfeeding (Massimino et al., 2019). We focused our work on the expression of genes involved in liver metabolism, thyroid pathway, cellular stress and cell proliferation at the end of the thermal treatment to study the short term impact, and before and after overfeeding for a longer term study.

## Materials and Methods

### Ethics Approval Statement

All experimental procedures complied with French national guidelines for the care of animals for research purposes. The protocols were approved by the Animal care and Use Committee of the Greater Southwest Region (no. 73) and authorized by the department under the file reference APAFIS14196-201805250850236-v3. Ducks were killed in line with the European Council regulation (European Union, 2009) at the Experimental Station for Waterfowl breeding (INRA, Artiguères, France).

### Eggs Incubation and Thermal Treatment

**FIGURE 1:**
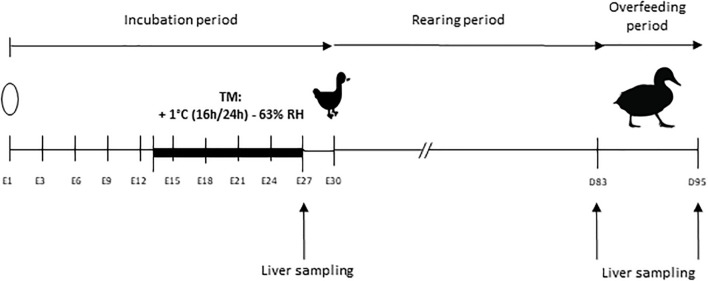
Experimental design. Incubation period extends from embryonic day 1 (E1) to day 30 (E30), day of peak hatching of the ducklings. The thermal manipulation (TM) was performed between E13 and E27, with a temperature increase of 1°C (16 h/24 h) and relative humidity (RH) adjusted to 63%. Overfeeding began at 83 days of age (D83) and ended at 95 days (D95). Liver samplings for RNA extraction are indicated by an arrow at the end of the TM (E27) and before (D83) and after (D95) overfeeding.

The overall experimental design is shown in Figure 1.

A total of 1,000 eggs of mule ducks (genotype H85, provided by Grimaud Frères Selection Company, Roussay, France) were kept at room temperature during 3 days prior to incubation and randomly divided into two incubators (500 eggs each). Control group was maintained at 37.6°C and 47% average relative humidity (RH) during the whole incubation period. Thermal manipulation (TM) was performed during the last 14 days of incubation period, i.e., embryonic day 13 to 27 (E13-E27) at 38.6°C 16 h/day (+1°C 16 h/24 h), RH being adjusted to an average of 63% in order to avoid egg dehydration. This thermal treatment condition was chosen among the three previously studied, for its optimal results, without any negative effect on hatchability or final yield (Massimino et al., 2019).

All eggs were turned through 90° every 3 h. In each incubator, temperature and hygrometry were continuously measured by a sensor equipped with remote probes (KIMO). Unfertile eggs were excluded by candling at E10, with a sliding of remaining eggs to prevent local temperature disturbance caused by the appearance of holes. On embryonic day 27 (E27), 20 embryos per group were collected and then temporarily placed in a small incubator near the dissection table, previously programmed to the incubation temperature, to avoid a long temperature variation before their slaughter by decapitation. Livers were harvested and frozen in liquid nitrogen for RNA extraction. The remaining eggs were placed in the same hatcher at 37.3°C and 80% of RH. Newly hatched ducks were recorded every day from E28 to E31.

### Rearing and Force-Feeding Conditions, Sample Collection

Male ducklings were divided into two groups of 70 per treatment and were raised under the same conditions of light and temperature, and fed *ad libitum* from hatching to 4 weeks of age with a starting diet (2,800 Kcal, crude protein 17.5%). From 4 to 8 weeks of age, ducklings were fed *ad libitum* with a growing diet (2,800 Kcal, crude protein 15.5%), and hourly rationed between 8 and 12 weeks of age. At 12 weeks of age (D83) 15 animals per group were slaughtered for liver sampling before overfeeding period, classically practiced at this age in ducks (Saez et al., 2010; Hérault et al., 2018; Lo et al., 2020). All remaining ducks were overfed with corn meal twice a day (53% corn and 47% water, Palma Maisadour), for 11 days (21 meals) and slaughtered at the end of the OF period (D95). Liver samples from 20 animals per group were collected for gene expression analysis, 10 h after the last meal. In each case, ducks were slaughtered by exsanguination after electronarcosis. After dissection, pieces of liver were sampled in the middle of the large lobe for the study of gene expression and were frozen in liquid nitrogen for RNA analysis.

### RNA Extraction and Reverse Transcription

Total RNA was isolated from frozen tissue according to the Ribozol method (VWR Life Science). Total RNA concentration was measured by spectrophotometry (optical density at 260 nm) using a Biotek EPOCH 2 microplate reader (Take 3 plate), and all the samples were normalized at 500 ng/μl. The integrity of total RNA was analyzed by electrophoresis, and the absence of DNA contamination was prevented by DNase treatment. Exogenous RNA of Luciferase (Promega) was added to each sample (100 pg per sample) to ensure the stability of a reference gene to enable data normalization, as described previously (Desvignes et al., 2011). For the RT-PCR, an amount of 3 μg RNA was reverse transcribed to cDNA with Iscript Reverse Transcription Supermix for RTqPCR (Bio-Rad, United States) with duplicate samples. Reverse transcription reaction was done in CFX384 (Bio-Rad, United States) according to this program: 25°C/5 min, 46°C/20 min, 95°C/1 min.

### qPCR EvaGreen Using BioMark

The mRNA levels of 45 genes encoding proteins involved in lipid and carbohydrate metabolism, cell stress and proliferation, and the thyroid hormone pathway were quantified in liver from ducks before and after OF (at 83 and 95 days of age). Given the large number of genes and samples, we chose for this part to do a high-throughput expression study. All primer sequences used are listed on the Supplementary Tables 1–3 and were validated on a concentration range of cDNA pool. Before performing Fluidigm step, a specific target amplification (STA) has been done with 5 ng/μL of cDNA. This step consists in PCR using PreAmp Master Mix and specific primer in order to normalize all samples and to ensure that there are enough copies of cDNA in each well. The manufacturer recommends this step because the method used nanovolumes. After the STA, all target and samples were distributed in a 96 × 96 chip for Fluidigm Gene Expression Array. The primers were provided by Applied Biosystems and were used at a concentration of 100 μM. The reaction was made using 20× EvaGreen dye following the program: holding stage 95°C for 10 min, 35 cycles of amplification: 95°C for 15 s, 60°C for 1 min. All the data were analyzed with the Fluidigm real-time PCR Analysis Software (Fluidigm Corporation v4.1.3). Melting curves were systematically monitored at the end of the last amplification cycle to confirm the specificity of the amplification reaction. Each qPCR run included negative controls (wells without reverse transcriptase, mRNA or cDNA). This part of the work was performed at the platform of quantitative transcriptomic GeT-TQ (GenoToul, Toulouse, France).

### Gene Expression Analysis After EvaGreen qPCR

The selectHKgenes function with “Vandesompele” method of the SLqPCR package was used with RStudio (Version 1.2.1335) to choose the 6 most stable housekeeping genes (Pfaffl et al., 2004). The six housekeeping genes for the relative quantification of mRNA levels of target genes were USP9X, beta-actin, EIF3, STAB1, HPRT1 and Luciferase (added during the reverse transcription step), and are listed in the Supplementary Table 3. The slope of a standard curve using serial dilutions of cDNA measured the efficiency (E) of PCR. In all cases, PCR efficiency values ranged between 1.85 and 2. The analyzes were done with RStudio and results were expressed as 2^−ΔΔ*Ct*^ with :


$$\mathrm{RQ}=2^{-\Delta \Delta \mathrm{Ct}}=\frac{{\text{RQ}}_{\text{target}}}{\text{geomean}\left({\text{RQ}}_{\text{references}}\right)}$$

$$\mathrm{RQ}=\frac{2^{\Delta {\mathrm{Ct}}_{\text{target}}}}{\begin{array}{c} (2^{\Delta {\mathrm{Ct}}_{\mathrm{USP9X}}}\times (2^{\Delta {\mathrm{Ct}}_{\mathrm{Actine}\beta }}\times 2^{\Delta {\mathrm{Ct}}_{\mathrm{EIF3}}}\times 2^{\Delta {\mathrm{Ct}}_{\mathrm{STAB1}}}\\ \times 2^{\Delta {\mathrm{Ct}}_{\mathrm{HPRT1}}}\times 2^{\Delta {\mathrm{Ct}}_{\mathrm{luciferase}}){}^{\frac{1}{6}}} \end{array}}$$

$$\Delta {\mathrm{Ct}}_{\text{target}}={\text{Ct}}_{\text{control}}-{\text{Ct}}_{\text{sample}}$$

$$\Delta {\mathrm{Ct}}_{\text{ref}}={\text{Ct}}_{\text{control}}-{\mathrm{Ct}}_{\mathrm{samples}}$$

$${\mathrm{Ct}}_{\text{control}}=\mathrm{average}\mathrm{Ct}\mathrm{of}\mathrm{all}\mathrm{samples}.$$

### Real Time qPCR Using Syber Green

Gene expression levels of embryonic liver samples (E27) were determined by real-time RT-PCR). Primer sequences are listed in the Supplementary Tables 2, 3.

The mRNA expression levels of target genes were detected using quantitative PCR with the PerfeCTa SYBR Green FastMix (Quantabio) in the CFX384 qPCR Detection System (Bio-Rad, United States). The reaction volume was 15 μL per sample and included 7.5 μL of SYBR Green FastMix, 2 μL (6 μg/mL) of cDNA and 2.75 μL of each primer (290 nM).

Melting curves were systematically monitored at the end of the last amplification cycle to confirm the specificity of the amplification reaction. Each qPCR run included duplicates of samples and negative controls (wells without reverse transcriptase, mRNA or cDNA).

### Gene Expression Analysis After Syber Green qPCR

Relative quantifications of target gene mRNA levels were normalized with exogenous luciferase RNA (Promega), constituting the reference gene (ref). PCR efficiency was measured by the slope of a standard curve using serial dilutions of cDNA. In all cases, PCR efficiency values ranged between 1.85 and 2. Relative quantifications were expressed as 2^−ΔΔ*Ct*^ (Livak and Schmittgen, 2001) with:


$$2^{-\Delta \Delta \mathrm{Ct}}=\frac{{\text{RQ}}_{\text{target}}}{{\mathrm{RQ}}_{\text{ref}}}$$

$${\mathrm{RQ}}_{\mathrm{target}}=2^{\Delta {\mathrm{Ct}}_{\left(\mathrm{target}\right)}}$$

$${\mathrm{RQ}}_{\text{ref}}=2^{\Delta \mathrm{Ct}\left(\mathrm{ref}\right)}$$

$$\Delta {\mathrm{Ct}}_{\text{target}}={\mathrm{Ct}}_{\text{control}}-{\mathrm{Ct}}_{\text{sample}}$$

$$\Delta {\mathrm{ref}}_{\text{target}}={\mathrm{Ct}}_{\text{control}}-{\mathrm{Ct}}_{\text{sample}}$$

$${\text{Ct}}_{\text{control}}=\mathrm{average}\mathrm{Ct}\mathrm{of}\mathrm{all}\mathrm{samples}.$$

### Statistical Analyses

Statistical analyses were done using the GraphPad Prism v6 software. For embryonic samples (E27), when data set presented a Normal distribution (assessed by Shapiro–Wilk test), student’s *t* tests were performed to compare control and TM groups. If the values did not follow a Normal distribution, the statistical analysis was performed with a non-parametric Mann Whitney test.

For pre-OF and post-OF samples, a two-way analysis of variance (ANOVA) was performed, followed by Sidak’s multiple comparison test. The data are presented as the average ± standard error of mean (SEM). In every case, differences between the groups were considered statistically significant if the value of *P* < 0.05.

## Results

### Expression of Heat Stress Pathway Genes at the End of the Thermal Manipulation

**FIGURE 2:**
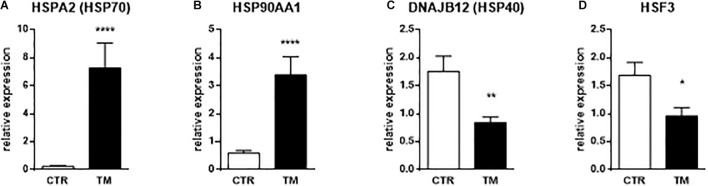
Hepatic relative expression of genes directly influenced by heat treatment at E27. Column representation of the relative expression of HSPA2 **(A)**, HSP90AA1 **(B)**, DNAJB12 **(C)**, and HSF3 **(D)** at the end of the thermal manipulation in the liver of control (CTR) or thermal manipulated (TM) duck embryos. Data are presented as mean +/– SEM (*n* = 18–19). *P < 0.05, **P < 0.01, ****P < 0.0001.

We first checked the expression of genes known to be influenced by heat stress to confirm that the thermal manipulation was indeed perceived by the liver of duck embryos. Relative expressions of three different heat shock proteins (HSP) and one heat shock factors (HSF) were analyzed in Figure 2 at the end of the TM (E27). We measured a strong increase in gene expressions of HSP70 and HSP90 in the TM group compared to the control group. In contrast, the expressions of DNAJB12 (HSP40) and HSF3 were significantly reduced in the TM group compared to the control group. These results confirm the molecular perception of the thermal stimulus by duck embryos.

### Impact of Early Thermal Manipulation and Overfeeding on Gene Expression of Metabolic Pathways

The Table 1 lists the relative expression values of genes involved in lipid and carbohydrate metabolisms. All these genes were significantly modulated by OF, with an overall increase in carbohydrate transport and oxidation, and lipid synthesis. The two main regulators ChREBP and PPARG were also increased at the end of the OF period compared to before this feeding challenge. On the contrary, lipid oxidation was strongly down-regulated after the last meal of the OF period compared to the pre-OF period. As shown in the right panel of the table, only two genes were significantly altered by the TM during this period: GPAT1 and APOB. The increase in GPAT1 expression was significantly higher in the TM group compared with the control group after the OF period (significant interaction between both factors), whereas APOB expression was decreased by both OF and TM, without significant interaction between both nutritional and incubation factors.

**TABLE 1:** Hepatic relative expression of genes involved in the lipid and carbohydrate pathways depending on incubation conditions before and after overfeeding.

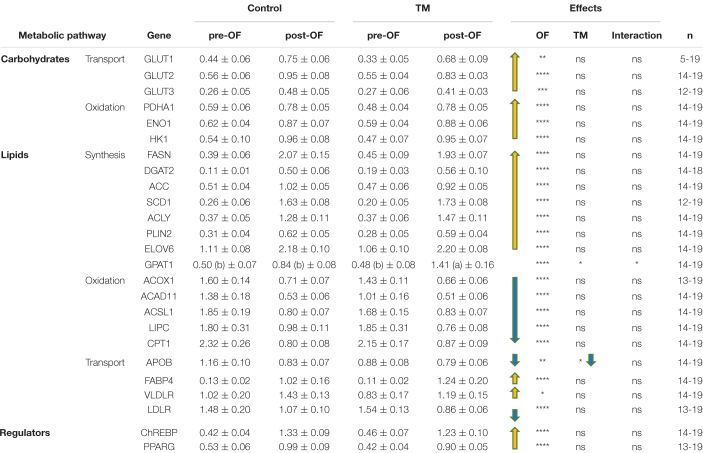

Data are presented as mean +/− SEM. TM, thermal manipulation; pre-OF, before overfeeding period; post-OF, after overfeeding period. ns, non significant, *P < 0.05, **P < 0.01, ***P < 0.001, ****P < 0.0001. Different letters in parentheses indicate significant differences measured between each group when there is an interaction between the OF and TM factors. n, number of samples.

### Impact of Early Thermal Manipulation and Overfeeding on Gene Expression of Thyroid, Cellular Stress and Proliferation Pathways

The Table 2 shows the relative expression of genes involved in various cellular pathways before and after OF in the liver. Most of these genes were significantly modified by OF. All profile types were represented in the thyroid pathway, with OF-induced increases in SPOT14 and THRB expression, decreases in NCOR and DIO3, and no OF effect for DIO1. Similarly, the cellular stress pathway was variously affected by OF, with an increase in HSP70, HSP90, HSF3 and ST13, and a decrease in UBQLN1, HSF2 and HSBP1 whereas PSMD12 were not altered at all by the nutritional challenge. The impact of OF on the cell proliferation pathway in the liver was more homogeneous with an increase in all relative expression values, except for HGF and MAPK1, which were not affected at all.

**TABLE 2:** Hepatic relative expression of genes involved in the thyroid, stress and cell proliferation pathways depending on incubation conditions before and after overfeeding.

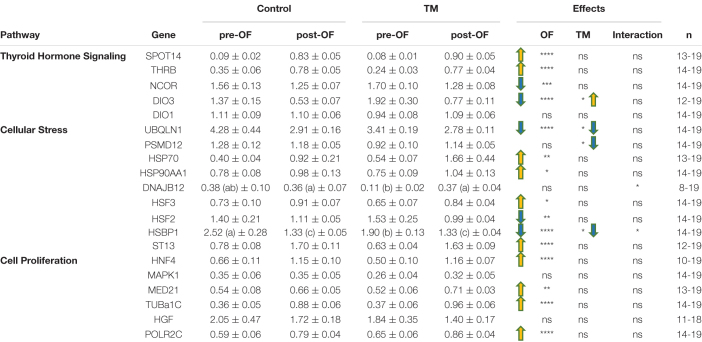

Data are presented as mean +/− SEM. TM, thermal manipulation; pre-OF, before overfeeding period; post-OF, after overfeeding period. ns, non significant, *P < 0.05, **P < 0.01, ***P < 0.001, ****P < 0.0001. Different letters in parentheses indicate significant differences measured between each group when there is an interaction between the OF and TM factors. n, number of samples.

None of the genes involved in cell proliferation were influenced by the TM unlike the stress pathway that involved two genes whose expression was significantly decreased by embryonic thermal treatment (UBQLN1 and PSMD12), and two for which a significant interaction between feeding and incubation conditions was found: it concerned DNAJB12 for which an increase was observed post-OF only in the TM group, and HSBP1, for which the decrease post-OF was most pronounced in the control group. Concerning the hormonal pathway, only the expression of DIO3 showed a significant increase in the TM group compared to the control group during this period.

### The Genes Influenced by Thermal Manipulation During Overfeeding Are Not Affected at the End of the Thermal Stimulus, Except One

**FIGURE 3:**
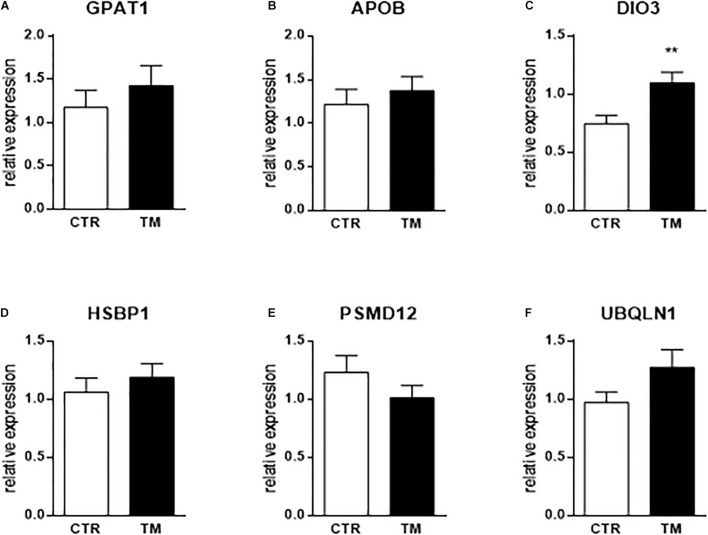
Hepatic relative expression at the end of the heat treatment (E27) of genes influenced by TM before or after overfeeding. Column representation of the relative expression of GPAT1 **(A)**, APOB **(B)**, DIO3 **(C)**, HSBP1 **(D)**, PSMD12 **(E)** and UBQLN1 **(F)** at the end of the thermal manipulation in the liver of control (CTR) or thermal manipulated (TM) duck embryos. Data are presented as mean +/– SEM (*n* = 16–19). **P < 0.01. The absence of a star means that there is no statistical difference between the groups.

As indicated above, only 6 genes (out of a total of 45) were significantly modulated by embryonic thermal treatment during the OF period. To see if this programming was already measurable at the end of the heat treatment, we measured the expression of these 6 genes in the liver of embryos at E27. The results presented in Figure 3 show that only DIO3 expression was already increased by TM at this stage, with the other five being not affected by the thermal treatment.

## Discussion

This study follows the demonstration that embryonic thermal programming can induce an optimization of liver fattening in mule ducks (Massimino et al., 2019). In this previous work, we used three different thermal stimuli during the incubation period to program the liver metabolism of adult ducks to respond more strongly to OF. Although all three programming resulted in larger and fatter livers than the control group, only one had no negative impact on hatchability or finished product quality. This condition consisted of a 1°C increase in incubation temperature, 16 h/24 h, from embryonic day 13 to embryonic day 27. We therefore chose to analyze the expression of genes in the liver of the animals from this condition in order to study the molecular mechanisms at the origin of this metabolic programming.

### Expected Molecular Perception of Heat Treatment at E27 Using Genes Known to Be Sensitive to Temperature

We began by studying the expression of genes belonging to the heat shock protein (HSP) family, themselves members of the chaperone protein superfamily (Morimoto, 1993; Vos et al., 2008). These proteins (including HSPA and DNAJ members) play a crucial role in stress conditions but also in normal physiological conditions requiring protein quality control for cellular homeostasis. Their binding to substrates at risk, i.e., in a degraded folding state, allows them to be directed toward a folding or degradation pathway. Although initially identified as heat stress-induced proteins, not all are regulated in the same way, and some not even by temperature (Vos et al., 2008).

In the first step of our experiment, we focused on three members of HSPs that are obviously altered by temperature: HSP70 (HSPA2), HSP90AA1 and HSP40 (DNAJB12). We measured a significant increase in the expression of the first two at the end of the heat treatment (E27) in the TM group compared to the control group, confirming what has already been well described in different tissues of heat-stressed chicken (Wang and Edens, 1998; Al-Zhgoul et al., 2013; Al-Zghoul et al., 2015; Cedraz et al., 2017; Albokhadaim et al., 2019; Madkour et al., 2021). More surprisingly, we also measured a significant decrease in HSP40 (DNAJB12) at E27 in the TM group compared to control. Mostly up-regulated after heat stress (Dong et al., 2006; Neal et al., 2006; Chen et al., 2018), down-regulation of that chaperone has, however, already been measured immediately after a transport stress in the spleen of hens (Li et al., 2021). These data suggest that the type of stress, the targeted tissue, or the time of stress exposure may have a different impact on the expression of different HSP family members.

Heat shock factors (HSFs) on the other hand are transcription factors induced after heat stress that bind to heat shock promoters to modulate the expression of target proteins (Westwood et al., 1991; Morimoto, 1993). HSF3 has been shown to regulate the production of HSP70 and HSP90 proteins under heat stress conditions (Tanabe, 1998). The decrease in its expression measured at the end of the heat treatment, in parallel with the strong increase of HSP70 and HSP90, could reflect the existence of a negative feedback after some time of heat exposure (14 days here), as already suggested in other types of stress responses and involving the glucocorticoid receptor (Wadekar et al., 2001, 2004; Kang et al., 2017).

However, all these changes in HSP and HSF gene expression confirm that the thermal stimulus used for embryonic programming was well perceived and measured at the molecular level in the liver of duck embryos.

### Molecular Impact of Overfeeding on Carbohydrate and Lipid Metabolisms but Also on the Thyroid Pathway, Stress and Cell Proliferation in the Liver

As explained above, this thermal embryonic programming has already been shown to increase *foie gras* production in ducks submitted to overfeeding between 83 and 95 days of age (Massimino et al., 2019). To understand the mechanisms behind this increase in fattening, we first explored the expression level of genes involved in lipid and carbohydrate metabolisms in duck livers before and after OF. As previously described, OF induced a strong increase in the expression of genes involved in carbohydrate transport to the liver (GLUT family), then in carbohydrate oxidation for pyruvate (HK1 and ENO1) and acetyl-CoA (PDHA1) production (Herault et al., 2010; Annabelle et al., 2017). These substrates can then be used by the pathway of lipid synthesis, strongly induced by OF in parallel with a decrease in their oxidation, as also observed previously (Tavernier et al., 2017; Annabelle et al., 2018). ChREBP and PPARG, key regulators of these pathways, are also strongly increased by OF. For lipid transport-related genes, the relative expressions of APOB and LDLR are decreased by OF, whereas those of FABP4 and VLDLR are increased. We propose two ways to explain these opposite regulations: first, depending on the direction of lipid transport through the liver cells (in or out), their expression can be either increased or decreased by the OF; second, the timing of RNA collection after the last OF meal can have a significant impact on the expression level, as demonstrated by a kinetic study of these same genes (which sometimes show opposite regulation at 2 h and at 5 h after the last meal) (Annabelle et al., 2018).

We then turned our attention to the thyroid hormone signaling pathway since it has been shown that, both in mammals (Danforth et al., 1979; Katzeff, 1990) and ducks (Massimino et al., 2019), OF induces an increase in plasma triiodothyronine (T3). The blood concentration in T3 is mainly dependent on the peripheral conversion of T4 in T3 by deiodinases. Especially, T4 can be converted into T3 by deiodinases 1 and 2, but into the inactive hormone rT3 by DIO1 and DIO3 (Darras et al., 2006). This active form T3 of thyroid hormone (TH) is able to bind a nuclear receptor (thyroid hormone receptor beta, THRB, being the predominant form in the liver) to modify the gene expression of a wide spectrum of genes involved in lipid metabolism (Sinha et al., 2018; Ritter et al., 2020). TH can induce the expression of fatty acid transporters, resulting in increased uptake of fatty acids in the liver, enzymes of lipolysis and beta-oxidation, but also *de novo* lipogenesis (Sinha et al., 2018). However, the circulating level of THs (T3 and T4) does not directly reflect their impact on tissue function, since they must first be captured by membrane receptors, then can be altered by deiodinases (DIO) and finally coregulators in the nucleus can regulate their activity (Mendoza and Hollenberg, 2017). Therefore, we were interested in measuring at the transcriptional level the hepatic regulation of their activity. Our expression data seem to confirm a global activation of T3 in the liver of overfed ducks, since we measured an increase in THRB and SPOT14 (involved in T3-induced lipid synthesis) expressions, in parallel with a decrease in NCOR (corepressor) and DIO3 (deiodinase responsible for TH inactivation) (Kinlaw et al., 1995; Mendoza and Hollenberg, 2017; Sinha et al., 2018; Ritter et al., 2020). This increase in T3 activity during overfeeding has been proposed as a protective mechanism to limit weight gain by increasing lipid catabolism in favor of energy expenditure through thermogenesis (Oppenheimer et al., 1991; Almeida et al., 1996; Silvestri et al., 2005). These regulations therefore suggest a protective mechanism for liver cells to limit OF-induced lipid overload, which may alter cell physiology over time.

To further assess the physiological status of liver cells during OF, we were then interested in the genes related to stress. We first notice that half of the chaperones involved in protein folding (HSP70, HSP90AA1, HSF3, ST13) are increased by OF, suggesting that protein quality control processes are globally activated. Decreased expression level of HSBP1 (in the control group, and to a lower extent, in the TM group) as an inhibitor of HSF (Cotto and Morimoto, 1999) could be interpreted as cellular protections. The decrease in HSF2 expression could, in another way, indicate that the transcriptional modulation of these factors does not always reflect their DNA-binding activity, as previously demonstrated (Sarge et al., 1991; Ding et al., 1996), although a negative feedback cannot be excluded in this global activation of protein protection. In another category, PSMD12 is a proteasome subunit that plays a role in global protein homeostasis by clearing misfolded or damaged protein (Du et al., 2020). Here, OF did not affect its expression, although it is downregulated in the liver of obese individuals with an increased fat content, as well as many genes involved in protein catabolism (Pihlajamäki et al., 2009). On the contrary, UBQLN1, also involved in protein degradation *via* proteasome or autophagy (Rothenberg et al., 2010; Kurlawala et al., 2017), is strongly decreased by OF, confirming at least a partial decrease in protein catabolism under these conditions.

Finally, since the role of hyperplasia in the fattening of duck livers after force-feeding has been recently put forward (Hérault et al., 2019), we measured the expression of genes involved in cell proliferation. Our results confirm a general increase in the expression of proliferation-related genes, with the exception of MAPK1, whose activation may be more reflected by the phosphorylation status (Nishida and Gotoh, 1993) and the hepatocyte growth factor (HGF) not affected at all. These observations suggest that hyperplasia may indeed play a role in duck liver fattening during OF, which should be further investigated by histological studies.

### Impact of Embryonic Thermal Manipulation During the Overfeeding Period – Evidence of Real Programming

Since ducks in the TM group had larger and fatter livers than controls at the end of OF (Massimino et al., 2019), we were first interested in the impact of TM on the expression of genes involved in the two main pathways directly implicated in liver fattening. Consistently with the study of Loyau et al. (2014) that had highlighted very low effect of TM in the hepatic expression of metabolic genes in the liver, none of the genes related to carbohydrate metabolism were significantly modulated by TM, and only 2 genes related to lipid metabolism were affected, and this at a relatively low level (*P* < 0.05), and only in case of overfeeding for GPAT1 (due to a significant interaction of the incubation and the nutritional factors for this gene expression). Located in the outer membrane of mitochondria, GPAT1 is involved in the first step of triacylglycerol esterification (Coleman, 2019) and has been shown to play critical role in hepatic steatosis (Yu et al., 2018). The second, APOB, is a structural protein of Very-Low-Density Lipoproteins (VLDL), and low-density lipoprotein (LDL) produced by the liver for lipid export to peripheral tissues (Gruffat et al., 1996; Bagherniya et al., 2021). Indeed, a greater increase in a crucial lipogenic enzyme or a decrease in a key factor involved in lipid export may both play a role in the increased liver fattening of our programmed animals. However, this relatively weak response led us to measure the impact of TM on the expression of other factors, possibly involved in liver metabolism.

Regarding the thyroid hormone signaling pathway, DIO3, considered to be the major TH-inactivating enzyme (Bianco et al., 2002), was significantly increased by TM before and after OF. This result contrasts with the study of Loyau et al. (2014) in the chicken liver who had found no change in this gene expression in slightly different conditions of TM. The expression of the activating enzyme DIO1 was not affected in both studies. The programming effect of TM on the regulation of the hepatic T4 to T3 or rT3 conversion might therefore be slightly different in the two species. Although no impact of thermal programming could be measured on plasma T3 and T4 levels in our overfed ducks (Massimino et al., 2019), increased expression of this deiodinase in the TM group suggests that embryonic thermal programming may induce a slight TH inactivation in the liver. These data support the hypothesis that embryonic thermal manipulation could have increased the lipid content in the liver by decreasing energy expenditure *via* thermogenesis (Almeida et al., 1996). Even though we did not measure a difference in surface temperature after the OF period in our programmed ducks, it is not impossible that internal temperature and thus thermogenesis was affected, reducing energy expenditure and thus promoting liver fattening.

Interestingly, the greatest impact of TM measured during the OF period was on the cellular stress pathway, with half of the significantly impacted genes belonging to this group. All of these genes were down-regulated in the TM group compared to the control group. Decreased expression of HSBP1, which is a negative regulator of the HSP family (Satyal et al., 1998), before OF, could be interpreted as a “protein quality control readiness,” preparing the liver to cope with OF and thus promoting improved cellular physiology during this challenge. TM also significantly decreases the expression of PSMD12 and UBQLN1, both involved in protein catabolism (Kurlawala et al., 2017; Du et al., 2020). As this metabolic pathway is decreased in the liver of obese patients with increased fat content (Pihlajamäki et al., 2009), this could represent a kind of predisposition to liver fattening.

Lastly, since it has been shown that embryonic thermal manipulation in chickens can stimulate cell proliferation in an immediate but also delayed manner (Piestun et al., 2009), we wondered whether TM could modulate liver cell proliferation during the OF period and thus contribute to liver fattening. Eventually, none of the genes we measured were affected by TM during this period. However, it might be interesting to measure the impact of TM on liver cell proliferation directly at the end of the thermal stimulus to see if baseline fattening potential is increased from birth by increasing cell numbers.

Finally, only six genes (out of 45) were significantly regulated by the embryonic TM during the OF period. However, these specifically targeted genes represent evidence of long-term programming at the molecular level in duck liver and may all support the hypothesis of optimized metabolism for *foie gras* production. Nevertheless, we do not rule out the possibility of having missed TM-induced transcriptional modulations during OF, especially because RNA samples were taken 10 h after the last feeding. It has been well demonstrated that the expression of metabolic genes during OF (Tavernier et al., 2017) but also according to the time of collection of liver samples after the last meal follow their own kinetics (Annabelle et al., 2017, 2018). Since most of the metabolic genes have a peak of expression about 2 h after the last meal, it would be interesting to study the impact of TM at this precise moment. In addition, the timing of the embryonic thermal stimulus may be optimized based on a recent study showing hepatic gene expression profiles during embryogenesis (Massimino et al., 2020), and may allow measurement of larger expression differences during the OF period. It is also important to note that all of these modulations are measured only at the mRNA level, and do not necessarily predict the activity of associated proteins, which is especially relevant for deiodinases for instance (Darras et al., 2006). It will be interesting during the next studies to confirm and deepen these results at the level of proteins and their activity.

### Genes Thermally Programmed to Respond Optimally to the Overfeeding Are Not Affected at the End of the Embryonic Stimulus, Except One

In the last part, we measured the direct impact of TM (at E27) on the six genes that were significantly impacted by long-term programming. We showed that the expression of only one of these genes was directly influenced by the TM at the end of the stimulus, suggesting that programming is registered through other pathways than direct modulation of expression. However, we observe that DIO3 is always up-regulated by TM both at the end of the thermal stimulus and before and after the OF period, suggesting a powerful and continuous mechanism to modulate this pathway which thus seems to have a key role in hepatic metabolism in ducks. Precisely how the expression of these genes is modulated by temperature, and how the information is recorded to program the response of hepatocytes to a dietary challenge much later in the life of the animal, however, are questions that remain to be explored.

## Conclusion

This study is the first to explore the molecular mechanisms involved in the long-term programming of liver metabolism in ducks by an embryonic thermal stimulus. We have identified a handful of genes that represent prime targets of this programming and may direct hepatic metabolism toward increased lipid storage during overfeeding. However, understanding the precise mechanisms behind programmed phenotypes will therefore require many more studies to be apprehended.

## Data Availability Statement

The original contributions presented in the study are included in the article/Supplementary Material, further inquiries can be directed to the corresponding author.

## Ethics Statement

The animal study was reviewed and approved by the Animal Care and Use Committee of the Greater Southwest Region (n° 73).

## Author Contributions

MH, SP, AC, MM, and WM conceived and designed the study. WM and CA conducted all the experiments and analyses with the help of SB, MH, TP, KR, KG, SD, and M-DB. M-DB supervised the whole breeding, overfeeding, and slaughtering phases. All authors reviewed the manuscript and approved the final manuscript.

## Conflict of Interest

The authors declare that the research was conducted in the absence of any commercial or financial relationships that could be construed as a potential conflict of interest.

## Publisher’s Note

All claims expressed in this article are solely those of the authors and do not necessarily represent those of their affiliated organizations, or those of the publisher, the editors and the reviewers. Any product that may be evaluated in this article, or claim that may be made by its manufacturer, is not guaranteed or endorsed by the publisher.
