# Supplementary figures and images for: Impacts of Embryonic Thermal Programming on the Expression of Genes Involved in Foie gras Production in Mule Ducks
Source: Front Physiol. 2021 Dec 3;12:779689. doi: 10.3389/fphys.2021.779689 (PMC8678469; doi:10.3389/fphys.2021.779689)

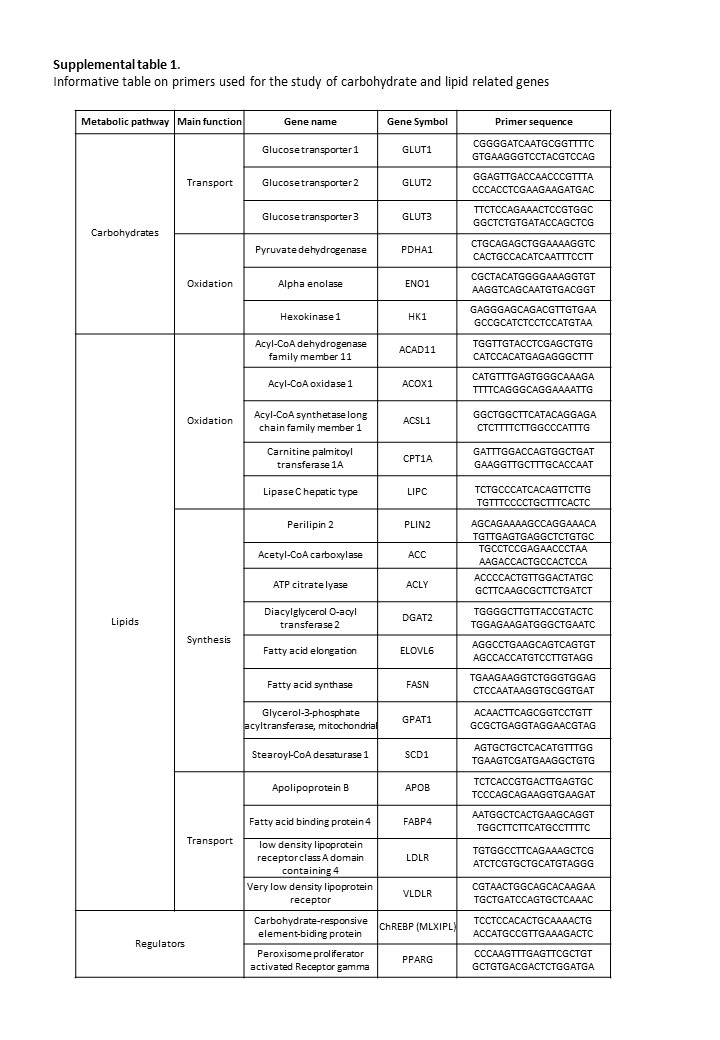

Supplement: Supplementary file 1 [file Image_1.JPEG]

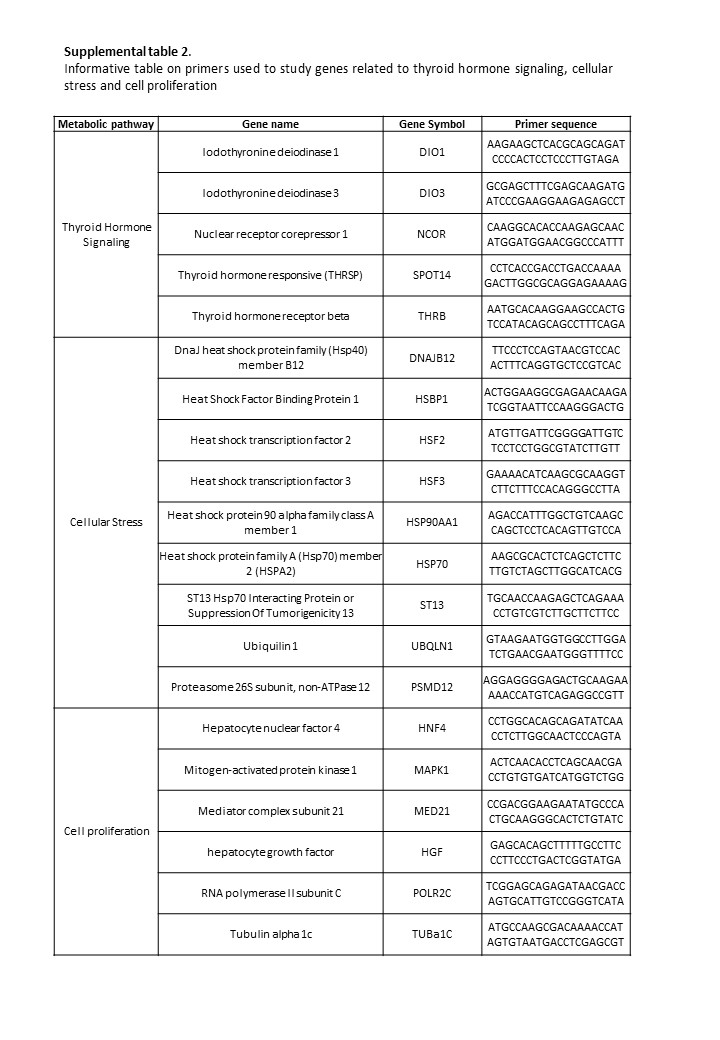

Supplement: Supplementary file 2 [file Image_2.JPEG]
