# Supplementary material for: Impacts of Embryonic Thermal Programming on the Expression of Genes Involved in Foie gras Production in Mule Ducks
Source: Front Physiol. 2021 Dec 3;12:779689. doi: 10.3389/fphys.2021.779689 (PMC8678469; doi:10.3389/fphys.2021.779689)
Supplement: Supplementary file 3 [file Table_3.pdf]

Supplemental table 3.

Informative table on the primers used for the reference geness

Gene name	Primer sequence
Actine beta	CCAGCCATCTTTCTTGGGTA<br>ATGCCTGGGTACATTGTGGT
EIF3	GATGAGGTGGCGGTGGATAT<br>TGGATTGGATTGTGCGCTTC
HPRT1	GTTGTGGGATACGCCCTAGA<br>ATGACAATGGGGGTGGATAA
Luciferase	CATTCTTCGCCAAAAGCACTCTG<br>AGCCCATATCCTTGTCGTATCCC
STAB1	CCTTGGAAACCCACAGAAAA<br>TACAGTCACCGCTGCTGAAG
USP9X	AGCCCTCTTCAGCAACTGTC<br>CCAAGCCATTCTACTGCCCA
